# Daily rhythms of diabetogenic factors in men: role of type 2 diabetes and body weight

**DOI:** 10.1530/EC-23-0064

**Published:** 2023-10-12

**Authors:** Cheryl M Isherwood, M Denise Robertson, Debra J Skene, Jonathan D Johnston

**Affiliations:** 1 Section of Chronobiology, Faculty of Health and Medical Sciences, University of Surrey, Guildford, United Kingdom; 2 Section of Metabolic Medicine, Food and Macronutrients, Faculty of Health and Medical Sciences, University of Surrey, Guildford, United Kingdom

**Keywords:** obesity, adipokine, diurnal rhythms, circadian, incretin

## Abstract

Obesity is a major cause of type 2 diabetes. Transition from obesity to type 2 diabetes manifests in the dysregulation of hormones controlling glucose homeostasis and inflammation. As metabolism is a dynamic process that changes across 24 h, we assessed diurnal rhythmicity in a panel of 10 diabetes-related hormones. Plasma hormones were analysed every 2 h over 24 h in a controlled laboratory study with hourly isocaloric drinks during wake. To separate effects of body mass from type 2 diabetes, we recruited three groups of middle-aged men: an overweight (OW) group with type 2 diabetes and two control groups (lean and OW). Average daily concentrations of glucose, triacylglycerol and all the hormones except visfatin were significantly higher in the OW group compared to the lean group (*P* < 0.001). In type 2 diabetes, glucose, insulin, C-peptide, glucose-dependent insulinotropic peptide and glucagon-like peptide-1 increased further (*P* < 0.05), whereas triacylglycerol, ghrelin and plasminogen activator inhibitor-1 concentrations were significantly lower compared to the OW group (*P* < 0.001). Insulin, C-peptide, glucose-dependent insulinotropic peptide and leptin exhibited significant diurnal rhythms in all study groups (*P* < 0.05). Other hormones were only rhythmic in 1 or 2 groups. In every group, hormones associated with glucose regulation (insulin, C-peptide, glucose-dependent insulinotropic peptide, ghrelin and plasminogen activator inhibitor-1), triacylglycerol and glucose peaked in the afternoon, whereas glucagon and hormones associated with appetite and inflammation peaked at night. Thus being OW with or without type 2 diabetes significantly affected hormone concentrations but did not affect the timing of the hormonal rhythms.

## Introduction

Many aspects of endocrinology and metabolism exhibit 24-h rhythms that are driven by an endogenous circadian timing system (1, 2, 3). This circadian system is comprised of a central light-entrained clock in the suprachiasmatic nuclei (SCN) of the hypothalamus and ‘peripheral’ clocks found elsewhere in the brain and throughout the rest of the body (4). Genetic lesioning of clocks in transgenic mouse studies has demonstrated the importance of individual peripheral clocks in key metabolic processes such as glucose homeostasis, insulin secretion and body weight regulation (5, 6, 7, 8, 9). These findings are likely to underpin long-standing observations that humans exhibit daily changes in post-prandial responses, glucose homeostasis and insulin sensitivity (10, 11, 12).

The SCN clock can synchronise peripheral clocks via a combination of neuronal, endocrine and behavioural pathways (e.g. the overt daily sleep–wake and feed–fast cycles) (13). Of these, feeding time is a dominant synchronising factor for many mouse peripheral clocks and some human metabolic rhythms (13, 14). Although circadian clocks are usually aligned to daily environmental changes, they can become desynchonised from the environment and from one another in cases such as shift work and jet-lag. This circadian desynchrony leads to metabolic disturbance further emphasising the close interrelationship between circadian and metabolic physiology (15, 16).

Relationships between obesity, type 2 diabetes mellitus (T2D) and circadian rhythmicity have been reviewed elsewhere (12). In one notable study, mice made obese by a high-fat diet exhibit reduced amplitude of circadian rhythms (17), although these effects may be due to diet *per se* rather than obesity (18). Other data from mouse models suggest that T2D in obese animals may associate with rhythms that are dampened beyond that observed in obese animals without T2D (19).

In humans, there is growing evidence to support the functional link between pancreatic clocks, insulin secretion and T2D (20, 21). In addition, obesity has been associated with reduced amplitude in the daily rhythms of circulating hormones, such as leptin (22) and ghrelin (23). Reduced morning clock gene expression has been reported in peripheral leukocytes of T2D patients compared to weight-matched controls (24). Moreover, a study of abdominal adipose tissue reported a reduction in the number of rhythmic genes in the abdominal adipose transcriptome of obese T2D patients compared to lean healthy controls (25). We have previously reported data from the lean healthy, overweight (OW) insulin sensitive and OW T2D men who provided samples for this study. In these individuals, there was no significant difference in plasma leptin or clock gene expression in adipose tissue from the upper gluteal region (26, 27). There were, however, significant differences in rhythms of plasma melatonin and metabolite concentration between the groups (27, 28). Some of these analytes exhibited more pronounced rhythmicity in the OW-insulin-sensitive or OW-T2D groups. Together, these data indicate that adiposity and T2D do associate with altered aspects of metabolic rhythmicity in humans, but the nature of that association is complex and depends upon the marker measured or the type of tissue sampled.

In this study, we used a highly controlled diurnal protocol (mimicking a ‘normal’ day–night cycle) to test the hypothesis that body weight and T2D are associated with altered average concentration and 24-h profiles of diabetes-related hormones. Hourly nutritional drinks were ingested within the wake/feeding period to remove the acute post-prandial effects of large meals.

## Materials and methods

### Ethical approval

A favourable ethical opinion was obtained from the Surrey Research Ethics Committee (08/H1109/117) and the University of Surrey Ethics Committee (EC/2008/82/FHMS/Fast-track). Written informed consent was obtained from all participants.

### Participants

The 25 male participants were assigned to one of three study groups according to their BMI and T2D. The lean group had a BMI of 19–25 kg/m^2^ without any of the European Group for the Study of Insulin Resistance (EGIR) criteria for insulin resistance (29), for more detail see Otway *et al.* (26). The OW group had a BMI of 25–35 kg/m^2^ without the EGIR criteria for insulin resistance. The OW T2D group had a BMI between 25 and 35 kg/m^2^ plus a T2D diagnosis. Fasting blood samples taken at screening measured HbA1c, insulin and glucose. Blood pressure, height, weight and waist circumference were also measured on the screening day, with health status and medical safety confirmed by their general practitioner.

Full details of the inclusion/exclusion criteria have been previously published (26). Briefly males aged between 40 and 65 years, with a regular sleep–wake cycle (sleep duration between 6 and 8 h) were recruited. The participants within the three groups (lean,* n* = 8, OW, *n* = 10 and T2D, *n* = 7) were matched for age and screening questionnaires (Horne–Östberg score, Epworth score and Pittsburgh Sleep Quality Index score) assessing sleep and chronotype with no significant differences between the groups (Table 1). One participant in the lean group was a smoker and was required to refrain from smoking for 1 week before the study. Two T2D participants were diet and exercise controlled (no medication) and five participants were treated with combinations of metformin, statins, ramipril and hypertension medication (two taking statins only, two on statins and metformin and one participant taking metformin only).

**Table 1:** Participant screening data for the lean, OW and T2D groups.

Variable	Mean ± s.e.m.			*P*-value			
	Lean (*n* = 8)	OW (*n* = 10)	T2D (*n* = 7)	All	Lean vs OW	OW vs T2D	Lean vs T2D
Age (years)	53.6 ± 2.1	50.8 ± 2.3	57.3 ± 1.6	0.13			
Waist (cm)	88.9 ± 2.3	105.9 ± 1.4	113.5 ± 3.2	5.17E-07	6.79E-06	0.03	2.28E-05
Weight (kg)	69.7 ± 3.0	93.8 ± 2.9	97.4 ± 5.4	5.37E-05	3.15E-05	0.53	4.98E-04
BMI (kg/m^2^)	23.2 ± 0.5	30.1 ± 0.8	32.0 ± 0.9	7.48E-08	2.35E-06	0.12	5.63E-07
Glucose (mM/L)	4.2 ± 0.2	4.8 ± 0.2	6.7 ± 0.5	1.31E-04	0.09	0.002	6.85E-04
Insulin (pM/L)	28.1 ± 5.9	40.6 ± 5.6	102.1 ± 30.6	0.01	0.15	0.03	0.02
HbA1c (mM/L)	5.4 ± 0.2	5.4 ± 0.1	6.9 ± 0.3	1.63E-05	0.93	1.43E-04	3.34E-04
BP systolic (mmHg)	129.4 ± 3.4	134.6 ± 3.4	147.3 ± 3.7	0.01	0.30	0.03	0.003
BP diastolic (mmHg)	84.0 ± 3.6	85.9 ± 3.3	89.6 ± 3.2	0.55			
HÖ score	59.0 ± 2.3	57.4 ± 2.8	59.7 ± 4.3	0.52			
Epworth score	3.0 ± 0.8	5.0 ± 0.7	5.6 ± 1.5	0.13			
PSQI score	3.4 ± 0.5	4.4 ± 0.6	3.7 ± 0.7	0.39			

Mean values ± s.e.m. and one-way ANOVA, followed by Tukey’s multiple comparisons test.

BMI, body mass index; BP, blood pressure; HbA1c, glycated haemoglobin; HÖ, Horne–Östberg; PSQI, Pittsburgh Sleep Quality Index.

### The pre-study protocol

Full details of the pre-study protocol are described in Otway *et al.* (26). Briefly participants followed a prescribed sleep−wake routine the week before the laboratory study to minimise the phase shifting effect of different sleep−wake times and the associated light−darkness environment on the circadian system. This included staying in bed from 22:30 h to 06:30 h (no deviation of more than 15 min) and obtaining at least 15 min of natural outdoor light within 90 min of waking. They were allowed to nap for up to 3 h, centred 12 h from the midpoint of their habitual night−time sleep (30) but had to avoid excessive exercise. The laboratory sessions were carried out between March and June 2009. Daily rest activity was monitored by actigraphy using light and motion monitors (Actiwatch-L, Cambridge Neurotechnology, Cambridge, UK) worn on the non-dominant wrist. Participants were required to call the laboratory voicemail when going to bed and getting up as well as keep a daily sleep diary.

During the pre-study period, meals were consumed within set time windows: breakfast 07:00−09:00 h, lunch 12:00−14:00 h and dinner 17:30−19:30 h. Food diaries were completed by the participants. Three days before the laboratory study only food provided by the study team could be eaten. Alcohol and caffeine were prohibited for the week before the laboratory session to avoid any influence on the endogenous timing system.

### The laboratory session

**Figure 1:**
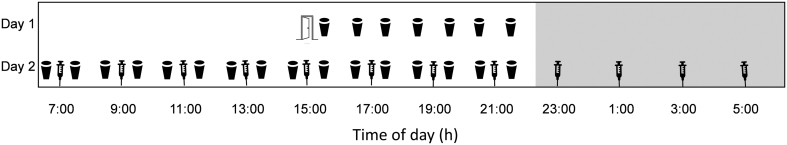
Participants entered the laboratory at 15:00 h on Day 1 (open door) and were housed in individual rooms. Lights on/wake occurred between 06:30 and 22:30 h, where light intensity was ~500 mW/m^2^ (160 lux) in the direction of gaze and 1600 mW/m^2^ (550 lux) on the horizontal plane. Participants lay semi-recumbent and were not permitted toilet visits during the 30 min period prior to the next blood sample. Participants lay recumbent during their sleep opportunity, which occurred in complete darkness (0 lux) between 22:30 and 06:30 h. Hourly milkshakes (FortisipTM) were administered from 15:30 h on Day 1 to 21:30 h on Day 2 during wake hours (cup). Blood samples were taken every 2 h (syringe) via indwelling cannula on Day 2.

Participants entered the laboratory at 15:00 h on day 1, illustrated in Fig. 1. Light was approximately 500 mW/m^2^ (160 lux) in the direction of gaze and 1600 mW/m^2^ (550 lux) on the horizontal plane during lights on (06:30−22:30 h). Participants lay semi-recumbent throughout the laboratory session to minimise postural effects. They were permitted to go to the toilet during waking hours but not within 30 min of the next blood sample. Sleep occurred in complete darkness (22:30–06:30 h), neither food nor toilet visits were permitted at this time.

To avoid acute effect of large meals, participants were given isocaloric milkshakes (Fortisip™ Nutricia, Schiphol, The Netherlands) with an energy density of 150 kcal/100 mL every hour, on the half hour, during waking hours. The first milkshake was given at 15:30 h on day 1 and the last milkshake was at 21:30 h on day 2. Water was provided *ad libitum*. To maintain body mass, the amount of milkshake was adjusted to each individual’s energy requirements (basal metabolic rate × 1.1, as participants were confined to bedrest), estimated using the Schofield equation (31).

Blood samples were collected every hour from 07:00 h on day 2 to 05:00 h on day 2 via an indwelling cannula inserted into the participant’s forearm vein by a trained nurse and immediately placed on ice. Plasma (di-potassium-EDTA) samples were stored at −80°C until the analysis was performed. Data for glucose and TAG for 23 of the 25 participants have been previously published (28).

### Metabolite and hormone assays

As described previously (28), plasma samples were assayed for glucose and triacylglycerol (TAG) using ILab 650 Clinical Analyser (Instrumentation Laboratory, North Risley, UK). The ILab was calibrated before every experimental run using standard ReferrIL G (Instrumentation Laboratory).

The hormones (insulin, C-peptide, glucose-dependent insulinotropic peptide (GIP), glucagon-like peptide-1 (total), glucagon, ghrelin, leptin, resistin, visfatin and plasminogen activator inhibitor-1 (PAI-1)) were measured with a multiplex immunoassay (Bio-Plex Pro™ Human Diabetes Assay, Bio-Rad Laboratories Ltd) requiring 50 µL of plasma per sample. The BioPlex software (version 6.1) automatically generated the standard curve using Brendan Scientific's 5-Parametric Logistic Regression. The analyses took place over nine separate runs. All the samples from each participant were measured in the same assay and the intra-assay % CV ranged from 2.5% to 13.2% (*n* = 3). Missing data from a combination of sources (missing samples, undetectable, outliers) were 5.6%. Each assay plate had at least one person from each of the three study groups.

### Statistical analysis

As per current laboratory methods of data processing, outliers were removed in the hormone data by removing any datapoint exceeding ± 3 s.d. from the mean of each participant time series.

Significant differences between study group, time of day and their interaction were determined using two-way repeated measures ANOVA (study group and time of day as factors) in R version 3.2.2 using the aov function in the stats package (32). Linear models were fitted to the study group and time of day (12 time points, repeated measures per individual), with the individual participant as covariate. *P*-values were corrected for multiple comparisons according to the Benjamini-Hochberg False Discovery Rate (FDR). Differences between variables were considered significant if the FDR-adjusted *P*-value <0.05.

Diurnal 24-h rhythmicity was assessed using cosinor analysis, using the group mean z-score values (z-score was calculated across each individual) (33). Peak time (acrophase), amplitude and significance of a cosine fit (*P* < 0.05) were determined (Matlab 2013b The MathWorks, Inc., Natick, MA, USA), using the cosine equation: *Y* = Mesor + amplitude × cos((2 × π × *X*/*T*) + phase) + residual.

## Results

### Differences in mean daily concentration between the groups

**Figure 2:**
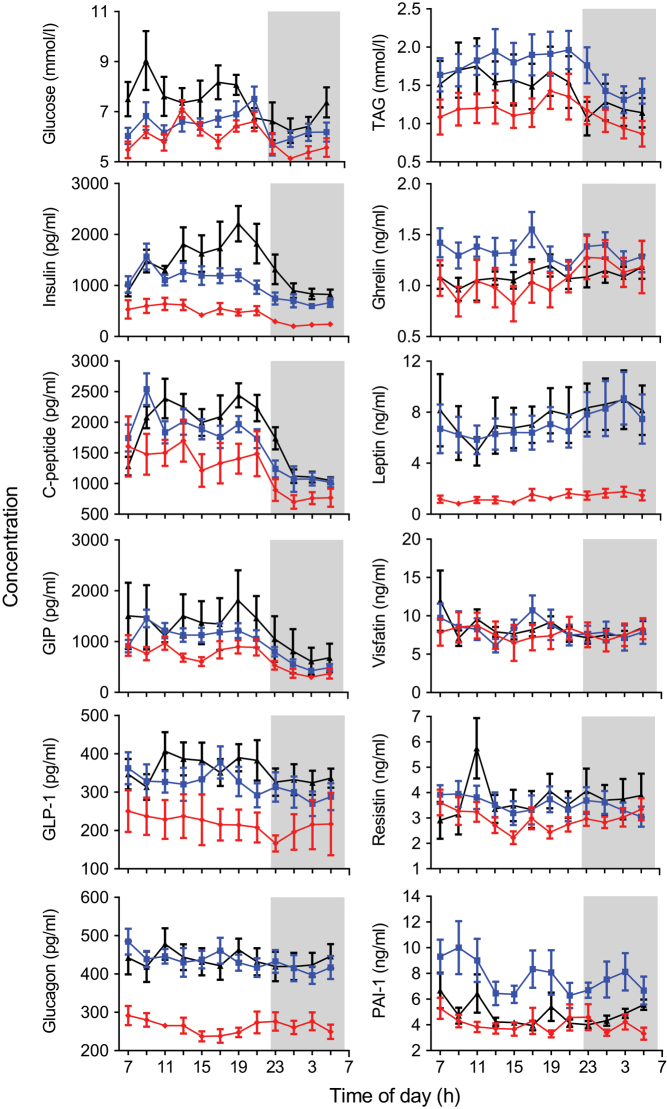
Mean (± s.e.m.) glucose, TAG and hormone concentrations of the lean (red), overweight (blue) and T2D (black) study groups across 22 h. Insulin, C-peptide, GIP, GLP-1, glucagon, ghrelin (pg/mL) and leptin, resistin, visfatin and PAI-1 (ng/mL) concentrations are displayed on the *y*-axis. Clock time is displayed on the *x*-axis, the sleep opportunity (22:30–06:30 h) is represented by the shaded area.

The glucose and TAG results have been published previously (28) but are replotted here with data from two additional participants included in this study but not in the published metabolomics work. The daily profiles of all ten hormones, glucose and TAG in the three study groups are presented in Fig. 2. Analysis by two-way repeated measures ANOVA, to assess the effect of increased body mass and T2D on hormone concentrations is reported in Table 2.

**Table 2:** Effect of study group and time of day on plasma hormone concentrations.

	All groups			Lean vs OW			OW vs T2D		
	Group (FDR)	Time (FDR)	Interaction (FDR)	Group (FDR)	Time (FDR)	Interaction (FDR)	Group (FDR)	Time (FDR)	Interaction (FDR)
Glucose	**9.5E-16**	**1.2E-08**	0.1	**5.4E-04**	**4.3E-07**	0.4	**1.5E-07**	**1.5E-05**	0.2
Insulin	**1.1E-42**	**7.2E-20**	**5.8E-05**	**2.2E-25**	**2.0E-11**	0.3	**3.3E-09**	**1.5E-14**	**2.9E-03**
C-peptide	**4.7E-12**	**6.2E-24**	0.04	**4.3E-07**	**2.9E-13**	0.5	**4.2E-02**	**2.2E-19**	**2.8E-02**
GIP	**9.8E-21**	**9.1E-28**	0.4	**7.0E-09**	**6.3E-18**	0.3	**1.8E-06**	**4.1E-18**	0.8
GLP-1	**9.0E-57**	**5.7E-06**	**1.9E-03**	**2.4E-33**	**2.1E-04**	0.1	**3.6E-07**	**3.7E-05**	**2.9E-03**
Glucagon	**1.9E-63**	0.2	0.4	**3.5E-46**	0.3	0.5	0.8	0.2	0.6
TAG	**1.2E-35**	**4.3E-16**	0.2	**6.3E-34**	**1.8E-11**	0.6	**4.7E-10**	**3.4E-12**	0.2
Ghrelin	**8.5E-15**	0.1	0.4	**1.8E-11**	0.2	0.3	**2.6E-08**	0.3	0.8
Leptin	**2.4E-65**	**3.0E-04**	0.9	**2.8E-53**	**8.3E-03**	0.5	0.1	**3.1E-03**	1.0
Visfatin	0.4	0.1	0.7	0.3	0.5	0.5	0.9	0.1	0.8
Resistin	**7.2E-06**	0.2	0.1	**3.2E-05**	0.3	0.7	0.3	0.3	0.2
PAI-1	**6.4E-17**	0.1	0.9	**7.6E-13**	0.4	0.7	**3.4E-08**	0.2	0.9

Values shown in bold indicate significant differences between the groups, time of day and group × time of day interaction (FDR < 0.05), two-way repeated ANOVA. An ANOVA was initially performed for all groups. Separate ANOVAs (the lean vs OW group and OW vs T2D group) were performed as the lean group was not directly comparable to the T2D group.

GIP, glucose-dependent insulinotropic peptide; GLP-1, glucagon-like peptide-1; PAI-1, plasminogen activator inhibitor-1.

The average daily concentrations of glucose, TAG and all the hormones were significantly higher in the OW group compared to the lean group (FDR < 0.001) except for visfatin (FDR = 0.3) (Table 2). Leptin exhibited the greatest change, with over a four-fold difference between the lean and the OW groups. Glucose, insulin, C-peptide, GIP and GLP-1 had significantly higher concentrations in the T2D group than in the OW group (FDR < 0.05, Table 2). By contrast, TAG, ghrelin and PAI-1 had significantly lower concentrations in the T2D group compared to the OW group (FDR < 0.001, Table 2). Glucagon, leptin, visfatin and resistin were not significantly different between the OW and T2D groups.

### Daily rhythms

In addition to daily concentration differences, ANOVA revealed a significant effect of time of day for glucose, insulin, C-peptide, GIP, GLP-1, TAG and leptin (Table 2). Insulin, C-peptide and GLP-1 displayed a significant interaction effect (group × time of day) between the OW and T2D groups. By contrast, there was no significant interaction (group × time of day) between the lean and OW groups (Table 2).

**Figure 3:**
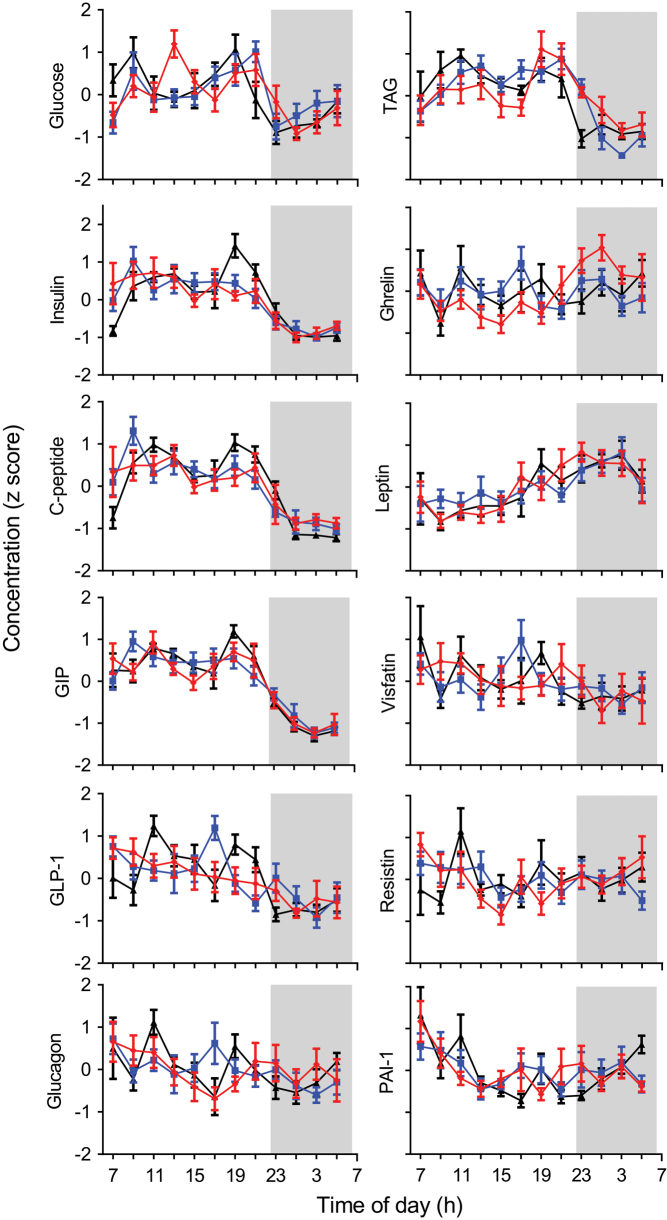
Mean (± s.e.m.) glucose, TAG and hormone normalised concentrations (z score) of the lean (red), overweight (blue) and T2D (black) study groups across 22 h, where each group z score was calculated from the individual z-score data. Normalised concentrations are displayed on the *y*-axis. Clock time is displayed on the *x*-axis, the sleep opportunity (22:30–06:30 h) is represented by the shaded area.

Figure 3 illustrates the diurnal rhythmicity of glucose, TAG and the hormones in the three study groups, using mean z-scored data to normalise for the inter-individual concentration differences and better reveal the shape of the daily rhythm. The goodness of fit of the group mean data to a 24-h sine wave was calculated using cosinor analysis. Glucose, TAG and all the hormones, except for visfatin, displayed a significant diurnal rhythm in at least one study group (Table 3). Insulin, C-peptide, GIP and leptin displayed significant diurnal rhythms in all three groups (*P* < 0.05). Glucose, glucagon, ghrelin and resistin displayed a significant diurnal rhythm in the lean group only (*P* < 0.05). GLP-1 displayed a significant diurnal rhythm in the lean and T2D groups (*P* < 0.05). TAG displayed a significant diurnal rhythm in the OW and T2D groups (*P* < 0.005) and PAI-1 displayed a significant diurnal rhythm in the T2D group only (*P* < 0.01).

**Table 3:** Cosinor analysis (acrophase, relative amplitude).

	Lean					OW					T2D				
	Acrophase time (dec. h)	Amplitude (*z*-score)	*P*-value	*n*	Individual cosine rhythms	Acrophase time (dec. h)	Amplitude (*z*-score)	*P*-value	*n*	Individual cosine rhythms	Acrophase time (dec. h)	Amplitude (*z*-score)	*P*-value	*n*	Individual cosine rhythms
Glucose	15.19	0.58	0.03	8	**2**			NS	9	3			NS	7	0
Insulin	13.06	0.70	0.01	8	**3**	**13.80**	**0.76**	**0.002**	10	**5**	**15.94**	**0.90**	**0.007**	7	**4**
C-peptide	13.65	0.61	0.02	8	**2**	**13.51**	**0.75**	**0.01**	10	**4**	**15.41**	**0.95**	**0.007**	7	**4**
GIP	**14.35**	**0.73**	**0.03**	8	**4**	**14.25**	**0.83**	**0.00**	10	**5**	**14.90**	**0.91**	**0.01**	7	**3**
GLP-1	11.86	0.51	0.01	8	**2**			NS	8	5	14.20	0.70	0.02	7	**0**
Glucagon	**2.92**	**0.47**	**0.02**	7	**5**			NS	9	2			NS	7	0
TAG			NS	8	1	**15.93**	**0.89**	**0.003**	8	**6**	**13.76**	**0.76**	**0.01**	7	**3**
Ghrelin	**1.74**	**0.69**	**4.6E-04**	8	**5**			NS	10	2			NS	7	0
Leptin	**23.54**	**0.73**	**2.2E-05**	8	**6**	**24.36**	**0.42**	**0.02**	10	**4**	**23.77**	**0.65**	**4.4E-04**	7	**3**
Visfatin			NS	7	4			NS	8	1			NS	7	1
Resistin	5.44	0.49	0.02	8	**1**			NS	10	2			NS	7	2
PAI-1			NS	8	1			NS	10	1	**7.35**	**0.69**	**0.01**	7	**3**

Bold text signifies a significant diurnal rhythm in the individual participant data (>40% of individuals). Underline signifies a significant diurnal rhythm in the individual participant data, not exhibited in the group mean.

GIP, glucose-dependent insulinotropic peptide; GLP-1, glucagon-like peptide-1; PAI-1, plasminogen activator inhibitor-1.

**Figure 4:**
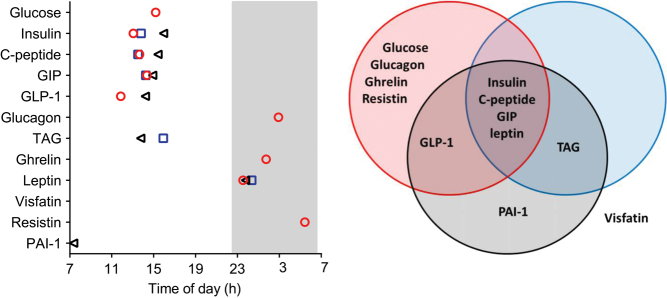
Peak time (acrophase) of hormones, glucose and TAG displaying significant diurnal rhythms. The analytes in the lean (red circle), overweight (blue square) and T2D (black triangle) groups are shown on the *y*-axis. The unshaded area illustrates the light/wake conditions (~160 lux in the direction of gaze, 06:30 h–22:30 h) and the grey shaded area illustrates the darkness/sleep conditions (0 lux, from 22:30 h to 06:30 h). Time of day (clock time) is displayed on the *x*-axis. The Venn diagram names analytes that had a significant cosinor fit in each group.

Analytes that displayed significant diurnal rhythms in more than one study group peaked at a similar time of day (within 3 h, Fig. 4). The hormones associated with glucose regulation (insulin, C-peptide, GIP and GLP-1) and TAG peaked in the afternoon, at a similar time to glucose; whereas glucagon, ghrelin, leptin, and resistin peaked in the darkness period and PAI-1 peaked in the morning (Fig. 4). Although rhythmicity was most commonly observed in the lean group, TAG and PAI-1 were only deemed rhythmic by cosinor fit in the OW and T2D groups, respectively (Fig. 4).

We finally assessed the goodness of fit to a 24-h sine wave of each individual within the study groups. Study groups with >40% of the participants displaying a significant cosine rhythm of glucose, TAG and the hormones are shown as bold text in Table 3. Only GIP and leptin displayed significant cosine rhythms in >40% of the participants in all study groups. Insulin, C-peptide and TAG displayed significant cosine rhythms in >40% of the participants in the OW and T2D groups. Significant cosine rhythms in >40% of the participants in a single study group were displayed by glucagon, ghrelin and visfatin (lean group), GLP-1 (OW group) and PAI-1 (T2D group).

## Discussion

We examined the impact of body mass and T2D on average daily concentrations and the diurnal rhythms of plasma glucose, TAG and ten hormones related to glucose regulation and inflammation under controlled sleep–wake and feed-fast conditions. The use of three experimental groups enabled us to identify the specific effects of body mass and T2D. Our study reveals novel information on the endocrine status related to increased body mass and T2D.

### Differences in mean daily concentration between the groups

As expected, being OW or being OW and having T2D was associated with higher concentrations of plasma glucose, TAG and many of the hormones. Except for visfatin, all the hormones had higher circulating concentrations in OW than in the lean group. The higher GLP-1 concentrations we observed in the OW group, compared to the lean group, are however not universally reported (34). There is also a considerable variation in studies depending on whether active or total GLP-1 has been measured and indeed which analytical method is used (35). GLP-1 is released very quickly upon feeding and, so it may be that the feeding protocol used here, where small feeds were given every hour has had a significant effect on the GLP-1 secretion (36) and degradation profile. Total GLP-1 levels have been shown to be highly dependent on the supply of duodenal nutrients, in T2D and in euglycaemia (37) and so it is likely that the high-energy fluid nutrients provided here were inducing a prolonged postprandial GLP-1 response. An additional interesting finding is the significantly higher GLP-1 levels in the T2D group compared to the matched OW group. The secretion of GLP-1 is usually considered to be inhibited in T2D; however, medications such as metformin have been shown to have a direct and AMP-activated protein kinase (AMPK)-dependent effect on GLP-1-secreting L-cells of the gastrointestinal tract (38). Due to the ubiquitous use of metformin as first-line therapy, metformin users were not excluded in this study.

Typically, circulating ghrelin is lower in obesity (39), contrary to our results where ghrelin was significantly higher in the OW vs lean group but significantly lower in T2D. However, it has also been shown that food ingestion fails to suppress ghrelin in obesity (40) which may have significant effects over a 24 h period on reported values. Although the protocol was designed to remove any significant postprandial peaks, the participants in this study were not being fasted and would have been provided with nutrients at a rate to suppress ghrelin secretion. The differential effects on ghrelin comparing T2D to OW participants are also of note. Deacylated ghrelin is known to have an additional, peripheral role in glucose metabolism, with low ghrelin levels associated with T2D independently from BMI effects (41). It needs to be noted however that the obese participants were selected based on them having normal insulin sensitivity, so in these BMI/adiposity matched groups, the low ghrelin is likely related directly to the impaired glucose metabolism.

### Daily rhythms

Daily rhythms of glucose metabolism and insulin sensitivity are well described (10). We and others have also characterised widespread diurnal and circadian rhythms of circulating metabolites in humans (28, 33, 42, 43, 44). However, daily rhythms of metabolic hormones remain poorly understood in humans, especially those who are OW and have T2D.

The data presented here support the argument that the effects of adiposity and T2D on daily rhythms are complex and must be considered in the context of specific metabolic pathways. Some mouse studies reported that obesity, T2D and high-fat diet result in reduced amplitude of diurnal rhythms (17, 24). In humans, gene expression in human blood cells (24) and abdominal WAT (white adipose tissue) (25) are consistent with lower amplitude rhythms in T2D. However, we have reported no amplitude differences in gluteal WAT gene expression (26) or plasma leptin concentrations (27). Furthermore, the nocturnal peak of plasma melatonin is significantly higher in obese, insulin-sensitive humans than lean controls (27, 45). Analysis of the human plasma metabolome revealed that some metabolites are rhythmic in obese and/or T2D humans but not in lean controls (28).

In this study, data from our multiplex hormone assay showed strong similarity to published radioimmunoassay and ELISA data from equivalent laboratory protocols. For example, leptin and ghrelin concentrations peak at night; in overweight individuals, leptin retains its nocturnal increase whereas ghrelin does not (23, 27). PAI-1 only displayed significant daily rhythms in the T2D group and peaked in the morning, as described previously (46, 47).

We observed different effects of body weight and T2D on diurnal rhythms of metabolic hormones. Some hormones (ghrelin, glucagon, resistin) exhibited a diurnal rhythm in lean participants that was absent in OW and T2D participants. However, other hormones (insulin, C-peptide, GIP, leptin) exhibited similar rhythmicity in the three experimental groups. Our data therefore support the notion that there is no simple relationship between obesity, T2D and presence of diurnal rhythms. Our data instead further support the notion that the interaction between metabolic status and 24-h rhythmicity varies between different metabolic pathways.

We examined the timing (phase) of the rhythms observed in our data. The hormones closely associated with glucose metabolism (e.g. insulin, C-peptide, and GIP) peaked in the afternoon, with peak times similar between the study groups. In the absence of food, under typical sleep–wake conditions, plasma insulin and C-peptide rhythms peak in the morning at 08:00 h (48) whereas, under constant routine conditions where individuals are kept awake in constant dim light with no overt feed-fast cycle, insulin peaks during the late biological night/early morning (49). The afternoon peaks in concentration observed in the current study therefore most likely relate to the accumulation of the hourly meals because the peak times for glucose and TAG were also in the afternoon.

This study is primarily concerned with the relative changes of concentration between groups and across 24 h. It is possible that the absolute concentrations measured in this single multiplex assay may vary from those derived in more commonly used assays. However, the consistency between the current data and comparable data derived from specific leptin radioimmunoassay (27) and ghrelin ELISA (50) provides external validation of these profiles.

## Conclusion

There are two main novel aspects of this study. The first is the use of tightly controlled conditions that remove large post-prandial effects; the second is the combination of three groups that enable separate analysis of body weight and T2D effects. Our data reveal previously unreported differences in endocrine rhythms associated with obesity and/or T2DM. The interaction between metabolic status and 24-h rhythmicity varies between hormones. It is possible that effects of medication in the T2DM group contribute to differences observed. The functional significance of these differently timed rhythms, including the relative contribution of endogenous circadian rhythms and daily behavioural patterns (e.g. medication, sleep–wake and feed-fast cycles) require further studies.

## Declaration of interest

The authors declare no conflict of interest that could be perceived as prejudicing the impartiality of the research reported.

## Funding

This research was funded by Diabetes UK http://dx.doi.org/10.13039/501100000361 (Grant 08/0003607), EU FP7-HEALTH-2011 EuRhythDia (Grant 278397), the UK Biotechnology and Biological Sciences Research Council http://dx.doi.org/10.13039/501100000268 (Grants BB/D526853/1 and BB/I019405/1) and Stockgrand Ltd. (Guildford, UK).

## Author contribution statement

JDJ designed the protocol and wrote the manuscript. CMI conducted the experiments, analysed the data and wrote the manuscript. MDR and DJS reviewed and edited the manuscript. JDJ is the guarantor of this work and had full access to all the data in the study and takes responsibility for the integrity of the data and the accuracy of the data analysis.
